# Smart respiratory monitoring: clinical development and validation of the IPI™ (Integrated Pulmonary Index) algorithm

**DOI:** 10.1007/s10877-016-9851-7

**Published:** 2016-03-09

**Authors:** M. Ronen, R. Weissbrod, F. J. Overdyk, S. Ajizian

**Affiliations:** 1 Medtronic, Jerusalem, Israel; 2 Medtronic, Boulder, CO USA; 3 Department of Anesthesiology, Roper St Francis Medical Center, Charleston, SC USA

**Keywords:** Respiratory compromise, Respiratory monitoring, IPI, Capnography, Composite index

## Abstract

Continuous electronic monitoring of patient respiratory status frequently includes PetCO_2_ (end tidal CO_2_), RR (respiration rate), SpO_2_ (arterial oxygen saturation), and PR (pulse rate). Interpreting and integrating these vital signs as numbers or waveforms is routinely done by anesthesiologists and intensivists but is challenging for clinicians in low acuity areas such as medical wards, where continuous electronic respiratory monitoring is becoming more common place. We describe a heuristic algorithm that simplifies the interpretation of these four parameters in assessing a patient’s respiratory status, the Integrated Pulmonary Index (IPI). The IPI algorithm is a mathematical model combining SpO_2_, RR, PR, and PetCO_2_ into a single value between 1 and 10 that summarizes the adequacy of ventilation and oxygenation at that point in time. The algorithm was designed using a fuzzy logic inference model to incorporate expert clinical opinions. The algorithm was verified by comparison to experts’ scoring of clinical scenarios. The validity of the index was tested in a retrospective analysis of continuous SpO_2_, RR, PR, and PetCO_2_ readings obtained from 523 patients in a variety of clinical settings. IPI correlated well with expert interpretation of the continuous respiratory data (R = 0.83, *p* <<< 0.001), with agreement of −0.5 ± 1.4. Receiver operating curves analysis resulted in high levels of sensitivity (ranging from 0.83 to 1.00), and corresponding specificity (ranging from 0.96 to 0.74), based on IPI thresholds 3−6. The IPI reliably interpreted the respiratory status of patients in multiple areas of care using off-line continuous respiratory data. Further prospective studies are required to evaluate IPI in real time in clinical settings.

## Introduction

Accurate assessment of patient respiratory status is an essential requirement of good patient care in all clinical settings: from pre-hospital and emergency care, through the spectrum of acute care within the hospital, and finally on the general medical surgical ward, respiratory status is a cornerstone of patient management. Spot checks of respiratory rate and SpO_2_ cannot provide a complete picture of respiratory status [4]. Continuous monitoring of oxygenation and ventilation using capnography and pulse oximetry allows providers to review trends in respiratory parameters not captured by intermittent monitoring, and promotes timely medical intervention that may prevent a respiratory arrest [1]. Recent publications have highlighted the added value of continuous capnography monitoring particularly when supplemental oxygen is being administered. Supplemental oxygen is often administered to patients undergoing procedural sedation and those receiving post-operative opioids, thus compromising the effectiveness of pulse oximetry monitoring alone in the timely recognition of respiratory insufficiency [2, 3]. The Anesthesia Patient Safety Foundation [4], American Society for Pain Management Nursing [5], and Joint Commission [3] recommend continuous monitoring of oxygenation and ventilation for patients at risk for respiratory compromise who receive opioids and sedatives [6], and the American Society of Anesthesiologists recommends the same for patients undergoing moderate to deep sedation calling for monitoring for the presence of exhaled carbon dioxide [7].

However, not all healthcare professionals are trained in interpreting four continuous channels of capnography and oximetry data, and ‘information overload’ may lead to erroneous conclusions rather than being helpful [8]. Research has shown that individuals have difficulty interpreting the overall significance of more than three parameters monitored concurrently [9]. In addition, high nursing staff turnover requires repeated training as new clinicians join a ward. There exists a need for simple, objective monitoring tools that may be easily deployed in hospital wards and do not require prolonged training to be used effectively.

We have developed a tool that simplifies the interpretation of continuous oximetry and capnography monitoring, allowing for expansion of this important monitoring modality into the ward space. The Integrated Pulmonary Index (IPI), an index score based on the integration of SpO_2_, PetCO_2_, RR and PR resulting in a single value representing respiratory status on a scale of 1 (critical respiratory insufficiency) to 10 (optimal respiratory status). This is the first commercially available tool incorporating ventilation and oxygenation into a single respiratory index score. Other indexes made use of vital sign parameters providing an early warning of patient deterioration and were developed using a “departure from normality” approach [11] as reported by Tarrasenko et al. In this report we present the design, development and validation of the IPI tool. We believe this to be the first example of a fused respiratory vital signs index based on implementing an expert rule system using fuzzy logic.

## Methods

### The IPI algorithm

IPI is a mathematical model that integrates four vital signs: end-tidal carbon dioxide (PetCO_2_), respiratory rate (RR), peripheral oxygen saturation (SpO_2_), and pulse rate (PR). A questionnaire was presented to 22 clinicians with expertise in respiratory monitoring, including nurses, doctors, anesthesiologists, and respiratory therapists. The questionnaire consisted of 85 combinations of the four parameters (PetCO_2_, RR, SpO_2_, and PR). The clinicians were asked to assign an IPI value from 1 to 10 to each combination of values, with a ‘1’ denoting ‘immediate intervention required’ to a ‘10’ denoting ‘no action required’ (Table 1).

**Table 1:**
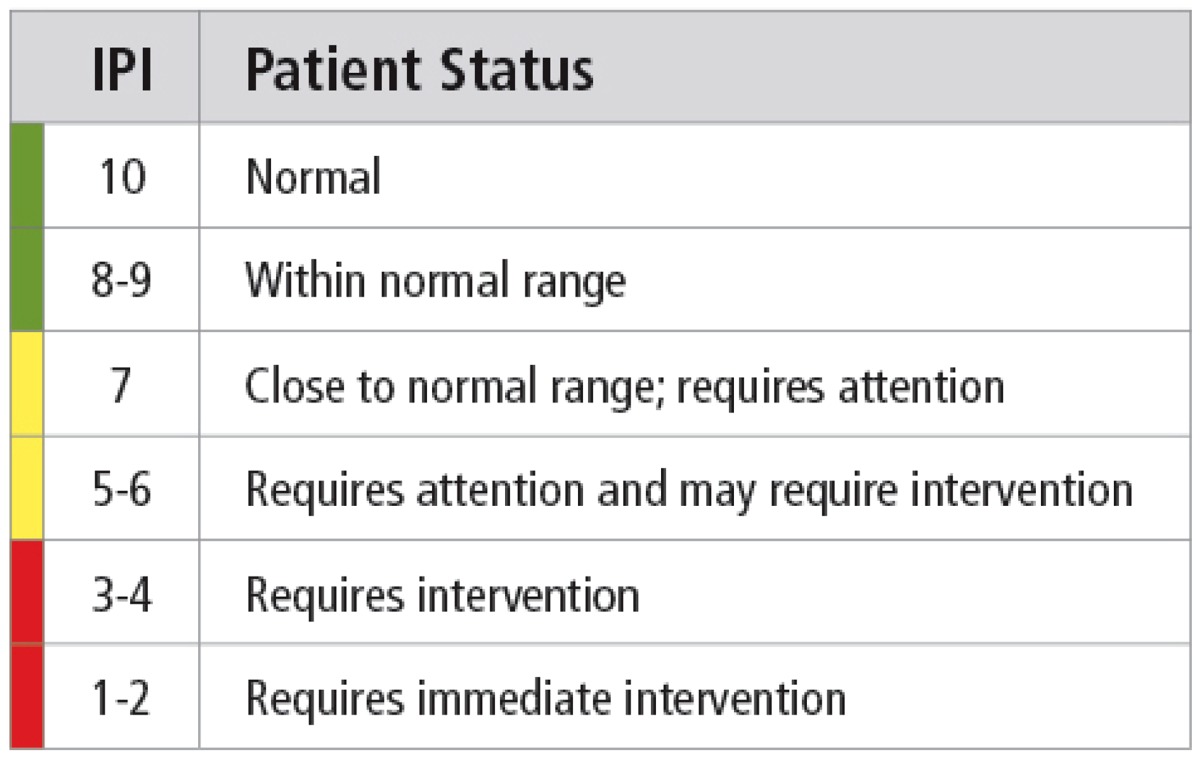
IPI Patient Status Descriptors

A fuzzy logic inference model [12] was then built using MATLAB^®^ Fuzzy Logic Toolbox™ software (Mathworks, Inc). Fuzzy logic is a model mimicking a human’s way of logical thinking, in that it deals with reasoning that is approximate rather than precisely deduced. Fuzzy logic uses verbal descriptors to define variables such as normal, high, low, and logical functions such as “If—then”, “Or” and “And”. Membership functions were assigned for each parameter based on the results of the clinician questionnaires and on commonly accepted tables in the literature. With these functions, vital-sign measurements were assigned membership in a set. For PR and SpO_2_, sets were high (H), normal (N), and low (L). For PetCO_2_ and RR, the sets were very high (VH), high (H), normal (N), low (L), and very low (VL).

**Fig. 1:**
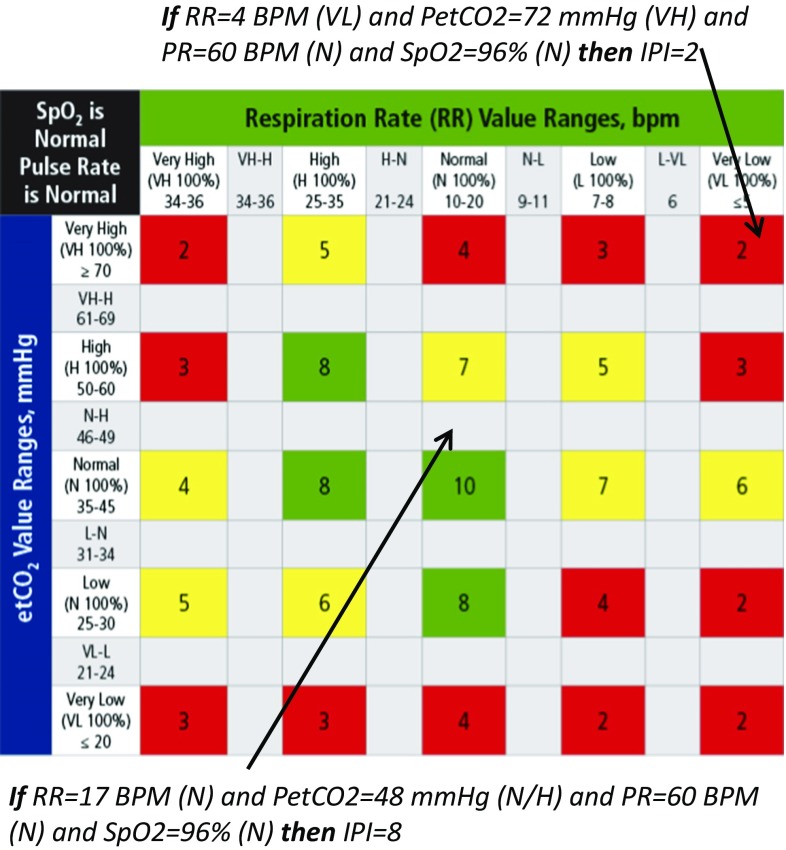
IPI algorithm rules. IPI is the intersection point of RR and EtCO_2_ values, assuming Normal PR and SpO_2_. *Gray areas* reflect partial membership in the adjacent range

**Fig. 2:**
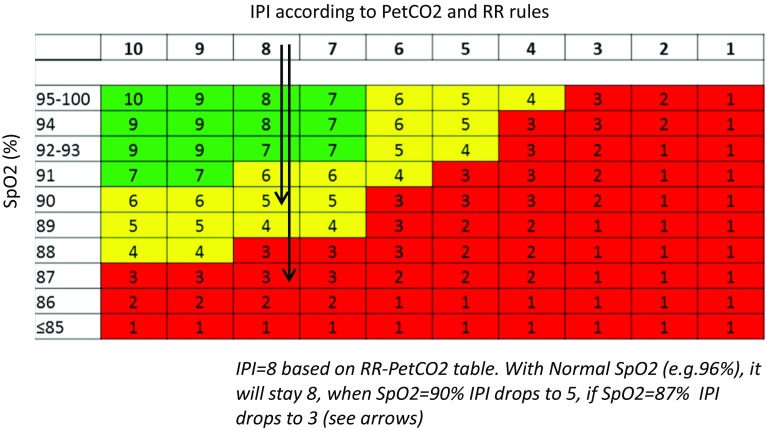
IPI algorithm rules—IPI values adapt to changing SpO_2_ patient values

A rule set was created to relate the inputs to an output matrix, using a verbal descriptor of the membership functions. For example, **if** (PetCO_2_ is VH) **and** (RR is VH) and (SpO_2_ is N) and (PR is H) **then** (IPI is 2). The rules are summarized in table format (Figs. 1, 2). The fuzzy logic operators used were: min for AND, max for OR, max for aggregation, and centroid of area for ‘de-fuzzification’. The fuzzy rules and membership functions were fine-tuned using an iterative process in which the experts were presented with additional sample cases and with the generated rules. The algorithm was designed as non-adaptive to patient pre-existing conditions or to trends over time providing absolute values requiring no customized setup by the clinician.

IPI is calculated by simultaneously evaluating the values of the four parameters. The final algorithm rules are illustrated in the following Figs. 1, 2. The rules matrix reflects the interactions between PetCO_2_ and RR and their effect on the IPI (Fig. 1). The effect of SpO_2_ is described demonstrating the decrease in IPI value as SpO_2_ decreases (Fig. 2). Pulse rates (PR) outside of the normal range will affect the IPI score as a secondary parameter as follows: If PR is L, and PetCO_2_ is H/VH, and RR is H/VH, then one is subtracted from the calculated IPI value. If PR is H, and PetCO_2_ is L/VL, and RR is L/VL, then one is subtracted from the IPI calculated value. All rules are applied simultaneously, yet the calculation of IPI can be demonstrated step by step. For example, in a case where RR = 12 BPM, PetCO_2_ = 26 mmHg, SpO_2_ = 90 % and PR = 70 BPM, we first use the table in Fig. 1, and assign IPI value according to the relations between RR and PetCO_2_, IPI = 8. Next we use the table in Fig. 2, where SpO_2_ effect on the previously calculated IPI is presented, to find that IPI drops from 8 to 5. PR does not affect IPI value in this example, according to the rule set.

#### Pediatric modes

In pediatric patients, normal vital sign ranges are age dependent. Three pediatric age ranges were used to calculate develop the pediatric IPI modes: ages 1−3 years, 3−6 years and 6−12 years. RR and PR membership functions were defined following literature review and discussion with eight pediatric experts (MDs and respiratory therapists) following the same methodology used for the adult data. The PetCO_2_ and SpO_2_ membership functions and the rules matrix were the same for adult and pediatric modes. IPI is not intended for children less than 1 year of age because the parameter ranges depend heavily on body weight and gestational age [13, 14].

### Model verification

A group of 22 clinicians independently scored 85 different combinations of the four parameters on the IPI scale from 1 to 10. We evaluated the correlation between the clinician derived IPI scores and calculated IPI scores using linear regression analysis and assessed the agreement between these measurements [bias (mean difference) and precision (standard deviation of the differences)] by the Bland–Altman technique for multiple observations [15].

### Reliability analysis

We hypothesized the IPI would recognize actionable respiratory events with a high level of sensitivity and therefore direct the clinician to rapidly assess the patient and intervene. The reliability of the IPI algorithm was evaluated against prospectively defined clinically significant events at different IPI thresholds. Events were divided into two categories for analysis: clinically significant events and severe events. The rationale of this approach was that not all respiratory events have equal importance and would trigger the same level of urgency in clinician response. Severe events were considered to warrant immediate intervention. Clinically significant events were considered actionable but not requiring immediate intervention. The goal of the analysis was to establish the sensitivity and specificity levels of the algorithm in detecting events that would trigger clinical interventions.

#### Event definition

Severe and clinically significant events were prospectively defined by clinicians based on published criteria for acute life-threatening events [16–19]:

**Severe events**
- Apnea—PetCO_2_ = 0 mmHg, RR = 0 bpm for at least 30 s.
- Severe hypoxia—SpO_2_ ≤ 85 % for at least 15 s.

**Clinically significant events**
- Central or obstructive apnea: PetCO_2_ = 0 mmHg, RR = 0 bpm for at least 15 s.
- Bradypnea and hypoventilation with hypoxia: PetCO_2_ > 50, RR < 8, SpO_2_ < 90 % for at least 15 s.
- Non bradypneic hypoventilation with hypoxia: PetCO_2_ < 30, RR 8−12, SpO_2_ < 90 % for at least 15 s.
- Hypoxia: SpO_2_ < 90 %, any PetCO_2_ and RR values for at least 15 s.

#### Data

Datasets of continuous recordings (sample rate at least 1 s) of PetCO_2_, RR, SpO_2_, and PR in a variety of clinical environments were used to create a clinical database (Table 2). This data was obtained from previous clinical trials in humans sponsored by the company and all had approval of ethics committees. IPI values were then calculated for each patient data set throughout the data recording. Inclusion criteria into the new database were the continuous recording of all four parameters for at least 10 consecutive minutes. This new database was then subjected to further analysis.

**Table 2:** Studies comprising clinical data used in the retrospective analysis

Studies comprising clinical database	Study description
1.	Quantitative and Qualitative Assessment of the Frequency and Validation of Alarms on the Alaris Medical PCA system with Oridion EtCO_2_ module, conducted at St Joseph’s Candler Health System, Savannah GA Principal investigator: Ray Maddox, PharmD Area of care: General Floor
2.	Smart Respiratory index—Clinical Evaluation Plan conducted at Hadassah University Medical Center in Jerusalem, Israel Principal investigator: Dr. David Gozal. MD Area of care: Pediatric Gastroenterology under procedural sedation
3.	Quantitative and Qualitative Assessment During an Upper Endoscopy Procedure of the Oxygen Delivery and EtCO_2_ sampling with the Smart Bite Bloc Mark III with oral O_2_ Delivery conducted at Bikur Holim Medical Center in Jerusalem, Israel Principal investigator: Prof. Samuel Adler Area of care: Adult Gastroenterology under procedural sedation
4.	Validation of the Oridion Capnography System in the PrehosPital and Emergency Department Setting Conducted at U.S. Army Institute of Surgical Research, Texas, USA Principal investigator: Dr. Jose Salinas, PhD Area of care: Adult trauma—EMS
5.	Smart ResPiratory index—Clinical Evaluation Plan (Hadassah), conducted at Hadassah University Medical Center in Jerusalem, Israel Principal investigator: Dr. David Gozal MD Area of care: Procedural Sedation
6.	Quantitative and Qualitative Assessment of the IPI set in Sha’are Zedek Medical Center, conducted at Sha’are Zedek Medical Center in Jerusalem, Israel Principal investigator: Prof. Yaacov Gozal MD Area of care: Post Anesthesia Care
7.	Comparison of the efficacy and safety of intravenous remifentanil PCA and epidural PCEA for labor analgesia. Conducted at the EPidural PCEA for labor analgesia, Jerusalem, Israel Principal investigator: Dr. Carolyn F Weiniger MD MRCA. Area of care: labor analgesia
8.	Capnography Library−data collection in the critical care environment, conducted at Shaare-Zedek Medical Center in Jerusalem, Israel Principal investigator: Dr. Sharon Einav MD Area of care: ICU
9.	A Pilot investigation to investigate the influence of CO_2_ sampling site on measured exhaled carbon dioxide during non-invasive pressure ventilation (NPPV)’. Conducted at Medical College of Georgia, Augusta, GA, USA Principal investigator: Arthur A. Taft, PhD., RRT Area of care: ICU
10.	Prospective Observational Clinical Trial to Investigate the Clinical Utility of the Integrated Pulmonary Index ™ (IPI™) to Predict Ability to Wean from Mechanical Ventilation’. Conducted at the Rush University Medical Center, Chicago, IL, USA Principal investigator: David Vines, MHS, RRT Area of care: ICU
11.	A Pilot investigation to investigate the influence of CO_2_ sampling site on measured exhaled carbon dioxide during non-invasive pressure ventilation (NPPV)’. Conducted at The University of Alabama at Birmingham, Birmingham, AL, USA Principal investigator: Arthur A. Taft, PhD., RRT Area of care: ICU

#### Analysis

Prospectively defined severe and clinically significant events as defined above were identified in the comprehensive recorded database. Similarly, we identified “IPI events” for each threshold of IPI (where IPI event is defined as IPI ≤ threshold value (1−9) for at least 15 s.). Then the continuous data was divided into 1-min epochs with overlap of 15 s. Epochs with events were identified and counted with the assumption of no interdependence of events. Sensitivity and specificity were calculated per each IPI threshold (Table 3), followed by receiver operating curve (ROC) analysis.

**Table 3:** Truth table used for calculation of sensitivity and specificity per IPI threshold

	Clinical event detected in epoch—*positive*	No-clinical event detected in epoch—*negative*
IPI event detected in epoch—*positive*	TP	FP
No-IPI event detected in epoch—*negative*	FN	TN

Sensitivity was calculated as TP/(TP + FN) and specificity as TN/(TN + FP)

## Results

### Model verification

**Fig. 3:**
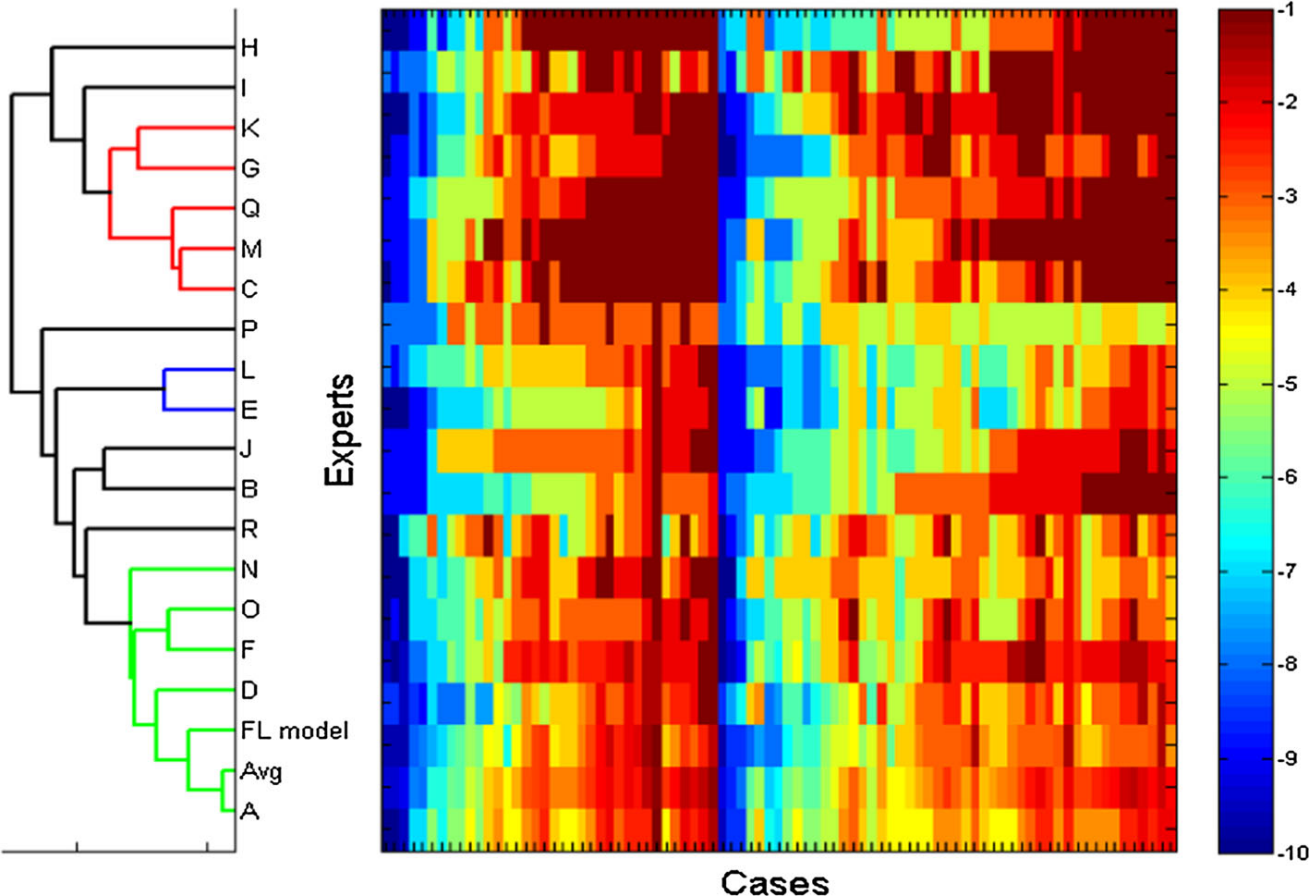
Cluster diagram showing the distribution of IPI value assignments for 85 cases by 18 medical experts reviewing the adult data, the average of their scores (Avg) and the fuzzy logic inference (FL model). *Columns* are cases, *rows* are expert or model. Color range: *blue* IPI = 10; *red* IPI = 1

The fuzzy logic inference IPI model was compared to experts’ IPI assessments (Fig. 3). The final model produced very similar results to those of the experts’ assigned IPI scores (Bias = −0.5, SD = 1.4) with high correlation (R = 0.83, *p* < 0.001). Across all experts and cases, the average absolute difference between experts and the model was 1.00 ± 0.35. Agreement between the model and the experts’ views is also apparent in the distribution of differences; where the absolute difference was less than 2 for 70 % of the data tested, and less than 3 for 92 % of the data.

### Reliability assessment

The database consisted of recordings of 556 cases and 33 were excluded due to short time duration. The remaining 523 cases constituted a total of 2563.3 h of valid patient data recordings with a median case duration of 1.5 h (0.2−45.2 h) per patient. The breakdown of the areas of care is presented in Table 4.

**Table 4:** Breakdown of clinical data by areas of care used in the retrospective analysis

Area of care	Number of valid cases
Post-operative analgesia^1^	18
Gastroenterology procedural sedation^2,3^	84
Trauma EMS^4^	94
Procedural sedation^5^	57
Post-anesthesia care unit^6^	43
Labor analgesia^7^	39
Intensive care unit^8−11^	188
Total	523

^1–11^The references to Table 4: The names of the clinical studies from which the clinical data used in the analysis was gathered

ROC analysis for detection of severe events at different thresholds of IPI demonstrated high sensitivity of IPI. At all thresholds tested, the sensitivity was above 0.99 (AUC = 0.995, CI 0.9994−0.9996, *p* ≪ 0.0001). The specificity decreased with the decrease in IPI threshold.

**Fig. 4:**
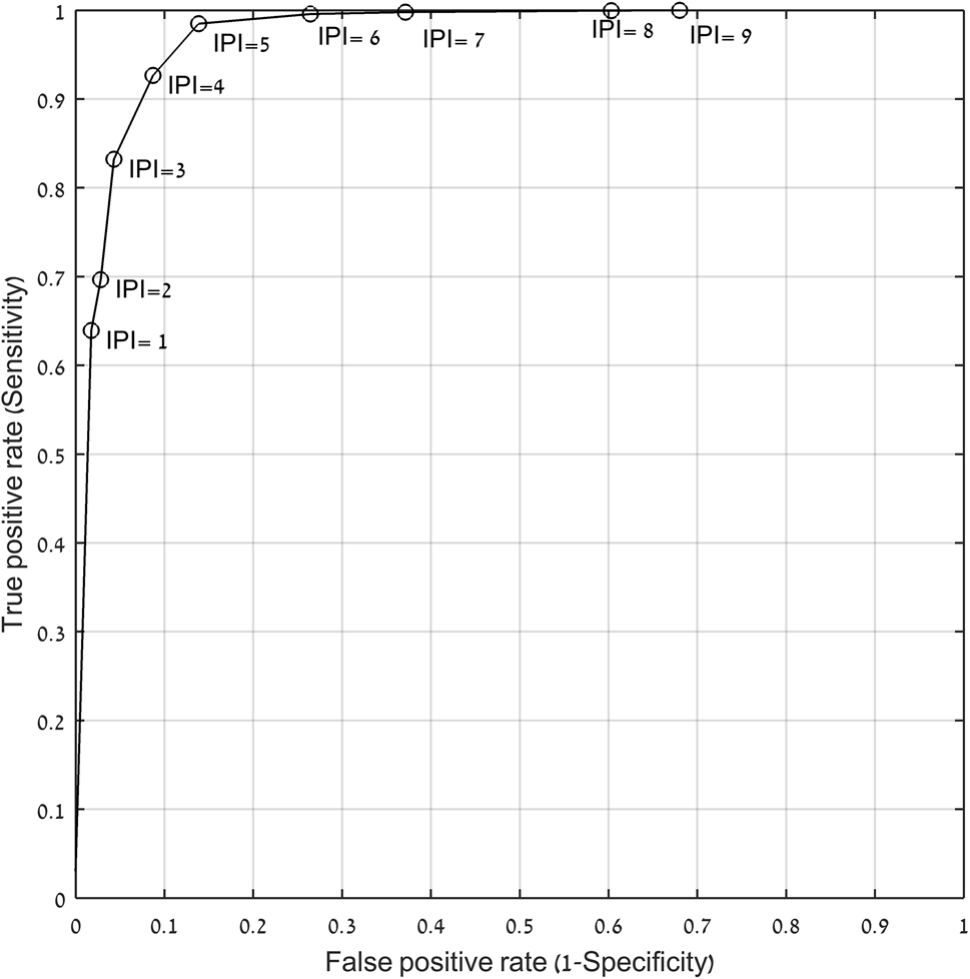
ROC plot for detection of clinically significant events using IPI (o—IPI threshold)

For the set of clinically significant events, ROC analysis (Fig. 4) demonstrated a very high sensitivity for IPI ≤ 6 (AUC = 0.977, CI 0.9767−0.9778, *p* ≪ 0.0001). At an IPI threshold of 3, sensitivity of 0.83 and specificity of 0.96 were calculated for clinically significant events. We evaluated false negative events at an IPI threshold of 3 and found that most of these events were transient hypoxia events in which short episodes of SpO_2_ values between 88 and 90 % occurred and were quickly resolved whenever hypoxic events progressed to severe events as defined above, the IPI recognized all events with a sensitivity of 1. The ROC demonstrates that the textual descriptors are well aligned with the actual performance of the algorithm. All severe and clinically significant events were correlated with low IPI values (Fig. 4).

The results demonstrate that the reliability of the algorithm in the detection of clinically significant events depends on the IPI threshold selected. At IPI settings of 3−6, a high level of reliability was demonstrated for both severe events and clinically significant events.

## Discussion

Continuous respiratory monitoring using both capnography and pulse oximetry can reduce the number of severe respiratory events and provide an early warning to clinicians when a patient’s status is deteriorating [1, 10, 16, 18, 20].

The analysis demonstrates high levels of sensitivity (0.83 for clinically significant events and 0.977 for severe events with IPI equal to or less than 3), for the IPI recognizing correctly clinically significant and severe events. In doing so, the IPI may be able to alert staff to patient events using a simple single digit algorithm, easy for the general ward staff to recognize. When patients experience pre-defined severe or clinically significant events, the index indicates that attention or immediate intervention is required. The textual descriptors presented in Table 1 provide appropriate guidance to the attending clinicians and could be particularly valuable for the less experienced clinician attending patients on a general ward.

The high specificity of the IPI for these events indicate the potential utility of the algorithm in preventing “cry wolf” scenarios, in which clinicians fail to respond to actionable device alarms due to high proportion of nuisance alarms from multiple devices in the hospital, with sometimes dire consequences [20]. When the IPI values are low, our analysis demonstrates the patient is experiencing a significant or severe respiratory event with a high level of probability. In cases when IPI values were equal to or lower than 3, yet the criteria for severe or clinically significant events were not met, we speculate whether these cases could still be indicating imminent deterioration due to the combination of changes in multiple parameters and this is a direction for future research. These cases may also highlight a known phenomenon of SpO_2_ being a lagging indicator of respiratory depression.

It is important to note that if clinicians select monitoring thresholds or definitions of events requiring intervention that are not consistent with the expert user definitions that were the basis for the algorithm assumptions, sensitivity will suffer [21]. This was the main reason the study by Berkenstadt et al., perceived lack of sensitivity, since they defined hypoxemia requiring clinician attention as SpO_2_ values below 92 % which is inconsistent with the algorithm. In the small scale study of 52 patients by Berkenstadt et al., no events requiring clinical intervention occurred which limits the applicability of this study for evaluating the general validity of the algorithm.

In our analysis we present ROC graphs for different IPI thresholds and distinguish between severe and clinically significant events, cognizant of the different needs across varying areas of care and patient types. Each institution would qualify the appropriate settings that would best address the clinical needs and the hospital policies the IPI alarm threshold should be set according to these principles. A future research direction would be the evaluation of the IPI as an alarm reduction tool through the use of the IPI alarm as a substitute for single parameter alarms.

We reiterate that although the reliability of IPI was tested using data streams from actual patients, the clinical events against which the algorithm was tested were not actual, but synthetically defined by the thresholds listed above for ‘clinically significant’ and ‘severe’ events. It stands to reason that the absence of CO_2_ for greater than 30 s on the capnograph should raise an alarm for an apneic patient. However, we have no clinical correlation from the site that the patient was actually apneic during the event. The sampling cannula may have been dislocated or removed. Thus the predictive values of the algorithm assume the signal is intact, free of noise, and accurately sampled and this may be considered a study limitation.

Respiratory events are a major cause of preventable deaths and simple tools to facilitate continuous patient monitoring and early identification of respiratory compromise are in need to address this challenge [1]. The IPI has been demonstrated to accurately detect and present respiratory events in a simple and clear manner and therefore could have a valuable role in improving respiratory monitoring practices. A potential application of this algorithm could be in the simplification of respiratory monitoring of post-operative patients receiving PCA analgesia as part of their treatment and may have value in controlling these pumps as part of a closed loop feedback system. These patients are typically found on the general wards in hospitals with the nurse being tasked with the care of multiple patients. Tools that may simplify the management of these patients and help prevent respiratory depression through a simple notification (e.g. IPI value fell beneath a certain threshold or a change in trend of IPI over time) are needed as hospitals seek to improve patient outcomes through prevention. Future studies will be required to assess the use as a predictive tool for early warning and the clinical utility of the IPI algorithm in different clinical environments including the application as a tool to reduce non-actionable alarms.

## Conclusions

The objective of the IPI algorithm is to simplify patient monitoring through real-time analysis of PetCO_2_, RR, SpO_2_, and PR, providing a single number that accurately indicates a patient’s respiratory status in a simple and objective manner. This simplification can extend the safety net of continuous respiratory monitoring far beyond the OR, ICU and PACU areas and onto the wards. The algorithm reflects the assessment of an expert group of clinicians.

The IPI accurately identified all prospectively defined severe events and identified clinically significant events at high levels of sensitivity and specificity.
